# The challenge of maintaining microscopist capacity at basic levels for malaria elimination in Jiangsu Province, China

**DOI:** 10.1186/s12889-018-5307-y

**Published:** 2018-04-12

**Authors:** Guisheng Ding, Guoding Zhu, Caiqun Cao, Ping Miao, Yuanyuan Cao, Weiming Wang, Yaping Gu, Sui Xu, Shengqiang Wang, Huayun Zhou, Jun Cao

**Affiliations:** 1 Nantong Center for Diseases Control and Prevention, Nantong, Jiangsu Province, People’s Republic of China; 2 Key Laboratory of National Health and Family Planning Commission on Parasitic Disease Control and Prevention, Jiangsu Provincial Key Laboratory on Parasite and Vector Control Technology, Jiangsu Institute of Parasitic Diseases, Wuxi, Jiangsu Province, People’s Republic of China; 3 Rudong Center for Diseases Control and Prevention, Nantong, Jiangsu Province, People’s Republic of China; 4 Wuxi Enter-Exit Inspection and Quarantine Bureau, Jiangsu Province, People’s Republic of China; 5 Public Health Research Center, Jiangnan University, Wuxi, People’s Republic of China

**Keywords:** Malaria elimination, Diagnosis, Microscopy

## Abstract

**Background:**

Local malaria transmission has decreased rapidly since the National Malaria Elimination Action Plan was launched in China in 2010. However, imported malaria cases from Africa and Southeast Asia still occur in China due to overseas laborers. Diagnosis by microscopy is the gold standard for malaria and is used in most hospitals in China. However, the current capacity of microscopists to manage malaria cases in hospitals and public health facilities to meet the surveillance needs to eliminate and prevent the reintroduction of malaria is unknown.

**Methods:**

Malaria diagnoses were assessed by comparing the percentage of first visit and confirmed malaria diagnoses at Centers for Disease Control and Prevention (CDCs) and hospitals. The basic personnel information for public health departments and hospitals at different levels was investigated. The skills of microscopists for blood smear preparation and slide interpretation were also examined at the county and township levels.

**Results:**

Inaccurate rate with 13.49% and 7.32%, respectively, in 2013 and 2014, from 341 and 355 reported cases from sub-provincial levels in Jiangsu province. Most of the 523 malaria cases reported in Nantong Prefecture from 2000 to 2014 involved patients who first visited county CDCs seeking treatment, however, none of these cases received confirmed diagnosis of malaria in townships or villages.The staff at county CDCs and hospitals with a higher education background performed better at making and interpreting blood smears than staff from townships.

**Conclusions:**

The network for malaria elimination in an entire province has been well established. However, an insufficient capacity for malaria diagnosis was observed, especially the preparing and reading the blood smears at the township and village levels, which is a challenge to achieving and maintaining malaria elimination.

## Background

**Fig. 1:**
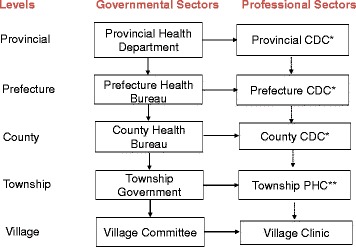
Public health system for malaria control and elimination in China. *CDC: Centers for Disease Control and Prevention; **PHC: Public health center in townships

**Fig. 2:**
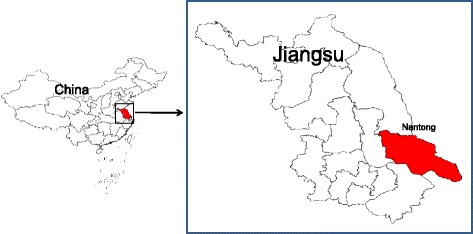
Study site in China

Remarkable progress has been achieved in China since the National Malaria Elimination Action Plan was launched in 2010, and reported malaria cases have declined rapidly; only 3116 malaria cases were reported in 2015 [1, 2]. However, the number of annual imported malaria cases has increased significantly in recent years because of increasing numbers of laborers and businessmen who work in Africa and other malaria-endemic areas [3, 4]. It is essential to detect all malaria cases in a timely manner for follow-up and foci treatment to prevent transmission or re-introduction. Timely diagnosis is also important prior to malaria treatment, especially for falciparum malaria, for which severe symptoms and death can occur without timely treatment with anti-malaria drugs [5, 6]. Furthermore, an accurate malaria diagnosis is crucial for the subsequent focal treatment to combat specific parasite species; this aspect is one of the most important issues for preventing secondary transmission and eliminating malaria in China [7]. After the severe global SARS epidemic in 2003 [8], CDCs from the national to lower levels in China were rapidly established and developed [9]. These CDCs replaced the former sanitation and anti-epidemic stations in China, and the current network for malaria control and elimination is based on this system (Fig. 1). Independent or combined departments from the national to lower-level CDCs (or institutes of parasitic diseases) are responsible for malaria control and elimination; in addition, hospitals at all levels in China are involved. All the CDCs and hospitals are managed under health departments from the national health and family planning commission of the People’s Republic of China (MOH) to the township governments. In this province, the regular refresher training courses are organized by both provincial and prefecture level every year to maintain the microscopical test skills from the majority of the counties’ level, and there are two mutual-checking microscopists meetings every year, in which the microscopists from all the 13 prefectures bring their positive and negative slides and checked by each other, in addition, the quality control system for the whole provincial microscopical test has been established, provincial reference lab for malaria diagnosis collect the slides quarterly and reviewed the reading accuracy and feedback the result to the administrative department. The number of rapid diagnostic tests (RDTs) that are available and the scale of their use for malaria detection has increased rapidly over the past few years; however, RDTs have a relatively poor detection rate for asymptomatic malaria cases with low parasite densities [10–12]. In addition, limited RDT products for malaria have been registered with the State Food and Drug Administration (SFDA) in China; therefore, microscopic examination is still the first choice for malaria diagnosis in most parts of China. In this study, malaria diagnosis in Jiangsu Province and a selected prefecture (Nantong) in central Jiangsu Province was investigated (Fig. 2). The blood smear preparation and interpretation skills of the staff at local public health departments were also assessed. Moreover, medical departments at the county and township levels were evaluated in 2013 and 2014 to identify surveillance challenges to malaria elimination and to prevent re-introduction.

## Methods

### Infrastructure investigation

Basic information, including the public health system network for malaria control and elimination, the number of malaria cases reported annually and the number of cases examined microscopically, was investigated in Jiangsu Province and Nantong Prefecture from 2000 to 2014. Furthermore, the educational background, work experience and number of personnel in public health departments (Centers for Diseases Control and Prevention, CDCs) and hospitals involved in malaria control and elimination at the county and lower government levels were investigated in Nantong Prefecture.

### Malaria diagnostic analysis

#### Initial malaria confirmation ability

The percentage of confirmed diagnoses for malaria among all of the reported malaria cases from 2013 to 2014 in Nantong were investigated, during the initial doctor visit due to febrile-related symptoms at different levels, i.e., the prefecture CDC, county CDC and prefecture hospitals at the county and township levels.

#### Assessment of blood smear preparation and interpretation

To assess operational ability of microscopy examination for malaria diagnosis, half of the township hospitals were randomly selected in Nantong, and one microscopist from each county CDC (8), county hospital (8), and selected township hospitals (36) was randomly selected to assess their skill at interpreting blood smears and tests to identify malaria parasites.Ten blood smears from febrile patients diagnosed as non-malaria cases at a CDC or hospital were investigated and scored. Ten indicators, including the blood volume, position, diameter, appearance, staining and clearance for both thick and thin blood smears, were scored based on criteria issued by the national CDC in 2011 to assess blood smear preparation and staining for malaria parasites [13]. One point was given for each of the 10 indicators (blood volume, position, diameter, appearance, stain quality and clearance for both thick and thin blood smears) for one slide, and ten slides were thoroughly checked. In addition, five blood smears comprising the four main malaria parasite species and a negative were randomly distributed on-site and tested by a microscopist or clinician. Each smear was microscopically checked for eight minutes and scored; 12 points were given for the correct determination of a positive or negative infection with parasites (20 points for negative slides), followed by 8 points for species identification. To ensure fair scoring, two experts from the provincial reference laboratory for parasitic diseases at the Jiangsu Institute of Parasitic Diseases who passed the diagnostic assessment for malaria parasites and obtained certification from the World Health Organization participated in the investigation. The average score of these experts was used to assess the preparation and staining of the blood smears.

### Statistical analysis

A chi-square test was used to compare the distribution of educational background, work experience and age between the county and lower levels in Nantong. An analysis of variance (ANOVA) test was used to compare the ability to accurately interpret parasites among the staff from county CDCs, county hospitals and township hospitals. ANOVA was also used to analyze the role that educational background, work experience and age played in the preparation of blood smears and the interpretation of parasites among the staff of county CDCs, county hospitals and township hospitals.

## Results

### Malaria in Jiangsu and Nantong from 2000 to 2014

**Fig. 3:**
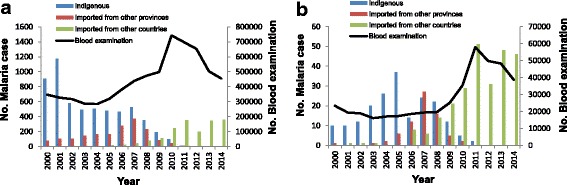
Malaria in Jiangsu Province (**a**) and Nantong Prefecture (**b**), 2000–2014. Columns of different colors show changes in the trend of cases imported from other countries (green), cases imported from other provinces (red) and indigenous cases (blue). The line (black) shows the change in the number of blood smear examinations

In total, 8359 and 523 malaria cases were reported in Jiangsu Province and Nantong Prefecture, respectively, from 2000 to 2014. Both Jiangsu Province and Nantong Prefecture exhibited a similar trend for malaria cases and blood examination. First, there was an obvious increasing trend of malaria cases imported from other countries after 2005, and 355 and 46 cases were reported in Jiangsu and Nantong, respectively, in 2014. Second, no indigenous malaria cases have been reported after 2011 in Jiangsu and Nantong. Finally, a steady increase in the number of annual febrile patients receiving blood examination was observed from 2000 to 2006 both in Jiangsu and Nantong, followed by a rapid increase from 2007 to 2011 and a decrease from 2012 to 2014 (Fig. 3).

### Malaria case diagnosis

In total, 341 and 355 malaria cases were reported by 13 prefectures in Jiangsu Province in 2013 and 2014, respectively. All the reported patient samples were sent to and rechecked by the provincial reference laboratory using both microscopy and PCR-based molecular methods. Overall, 46 (13.49%) and 26 (7.32%) cases, including all the four major human parasite species and mixed infections, in 2013 and 2014, respectively, were corrected. With an exception of *p.m* in 2014, a relatively low reporting accuracy was exhibited for both the *p.v* and *p.o* species in these two years (Table 1).

**Table 1:** Malaria parasite species reporting and correction in Jiangsu Province

2013							2014						
Reported*		Corrected**					Reported*		Corrected**				
		*P.f*	*P.v*	*P.m*	*P.o*	Mix			*P.f*	*P.v*	*P.m*	*P.o*	Mix
*P.f*	299	284	3	0	6	6	*P.f*	294	283	0	2	7	2
*P.v*	18	0	5	4	9	0	*P.v*	14	2	1	1	10	0
*P.m*	7	1	0	5	1	0	*P.m*	10	3	0	6	1	0
*P.o*	14	0	0	0	13	1	*P.o*	33	1	3	1	27	1
Mix	3	1	0	0	1	1	Mix	4	3	0	0	1	0
total	341	286	8	9	30	8	total	355	292	4	10	46	3

*: All of the malaria cases were diagnosed using microscopic examination or the RDT method in sub-provincial CDCs, hospitals and PHCs and were reported to the Jiangsu provincial malaria department through the web-based China Information System for Disease Control and Prevention (CISDCP) within 24 h

**: All of the blood samples in the reported malaria cases were rechecked using both microscopic examination and a PCR-based method in a provincial malaria reference laboratory

**Fig. 4:**
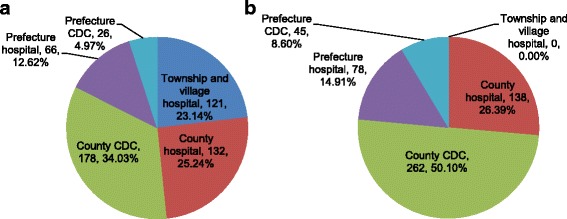
The distribution of malaria patient treatment and confirmation diagnosis in Nantong, Jiangsu Province. (**a**) refers to malaria patients who first sought treatment for febrile-related symptoms, and (**b**) refers to the confirmation of malaria infection

Almost half of reported malaria cases from 2000 to 2014 in Nantong involved patients who sought a diagnosis in the county hospitals (25.24%) or lower township and village hospitals and clinics (23.14%) because of malaria symptoms. Most of these patients went to the county CDCs (34.03%), and only a small percentage of patients visited the upper prefecture hospital (12.62%) or CDCs (4.97%). Similarly, most of the confirmed diagnoses occurred in county CDCs (50.10%), followed by county hospitals (26.39%), the prefecture hospital (14.91%), and the prefecture CDC (8.60%). There were no cases with a confirmed malaria diagnosis in the lower township hospitals or village clinics (Fig. 4).

### Microscopists in counties and townships

There was a total of 19 and 168 microscopists involved with malaria diagnosis in 8 county CDCs and 88 township public health centers (PHCs), respectively. There was a significant difference (X^2^ = 10.35, *P* < 0.01) between the county and townships according to the distribution of educational backgrounds, and more county microscopists had bachelor degrees (47%) than those in the townships (20%). However, there were no differences in terms of work experience (X^2^ = 3.72, *P* > 0.05), and most of the staff from the counties (47%) and townships (51%) had been working less than 5 years (Table 2).

**Table 2:** The distribution of educational background and work experience among microscopists and clinicians from different levels

	Educational background				Work experience (years)			
	Specialized secondary school	College diploma	Bachelor’s	< 5	5~ 10	10~ 15	15~ 20	> 20
County CDC	9	1	9	9	3	0	3	4
Township PHC	81	54	33	85	13	19	23	28

### Capacity of microscopists at a basic level

In total, 52 microscopists from county CDCs (8), county hospitals (8), and township hospitals (36) were selected for on-site assessment and testing. The staff from both county CDCs (mean = 90.00, *P* = 0.000) and county hospitals (mean = 82.50, *P* = 0.001) exhibited a higher score than the staff from township hospitals (mean = 57.94) for blood smear interpretation. Staff with a bachelor degree exhibited a higher blood smear interpretation score than staff without a higher degree (*P* = 0.015; *P* = 0.007). Furthermore, microscopists aged 40 to 50 years had better blood smear interpretation skills than staff younger than 30 years old (*P* = 0.044). For blood smear preparation, staff in township hospitals with a bachelor degree performed better than staff with specialized secondary school degrees (*P* = 0.042). In addition, staff who were 30 to 39 (*P* = 0.032) and 40 to 49 (*P* = 0.006) years old performed better than staff who were older than 50 years (Table 3).

**Table 3:** A comparison of blood smear preparation and interpretation capacity among the staff from different levels

		Number of microscopists			Average scores	
		County CDC	County hospital	Township hospital	Interpreting smear	Making smear
Educational background (degree)	Bachelor’s	3	3	4	84.00	98.03
	College diploma	1	0	12	62.15	94.00
	Specialized secondary school	4	5	20	62.69	87.99
Age (years)	< 30	0	0	7	54.57	92.83
	30–39	1	2	10	70.46	93.46
	40–49	4	2	7	75.67	97.00
	> 50	3	4	13	63.00	85.39
Work experience (years)	< 5	2	4	19	66.88	89.93
	6~ 14	2	4	4	66.00	92.18
	> 15	4	0	13	66.71	92.17

## Discussion

Jiangsu Province has had especially high malaria transmission in the last century; the malaria cases once reached more than 10 million a year, which is almost one-fourth of the total population of the entire province [14]. After concerted efforts from the national to regional levels, malaria transmission has been well controlled recently, and only hundreds of malaria cases have been reported in Jiangsu annually [15–17], despite fluctuations after a re-emergence of vivax malaria in central China from 2004 to 2006 [18]. No additional local malaria cases have been observed and reported since 2011; however, the total number of malaria cases increased markedly in the last several years because of oversea laborers who export and trade in China but have contact with areas of endemic malaria [4]. Nantong Prefecture was selected in this study because it shows a pattern of malaria spread that this similar to that of the entire province and is a good current representative.

The “1–3-7” strategy for the surveillance of and response to malaria elimination was produced by Jiangsu Province and was recently adopted as the national policy for malaria elimination in China [19, 20]. This strategy is defined as the reporting of malaria cases within one day, their confirmation and investigation within three days, and the appropriate public health response to prevent further transmission within seven days. Reporting of information is the first and one of the most important steps for malaria elimination. The correct diagnosis, including malaria infection and detailed parasite species classification, plays very important roles in case verification and focal treatment. A misdiagnosis of a malaria species might lead to the use of inappropriate antimalarial drugs and secondary transmission by local malaria vectors in the absence of timely vector control measures such as IRS activity [21]. In this study, a relatively low reporting accuracy was found in terms of the *P.v* species from 13 prefectures in Jiangsu Province, and *Anopheles sinensis*, one of the most effective malaria vectors, especially for transmitting vivax species in China, is widely distributed in the entire province [22]. When the infection source accumulates without the correct foci treatment due to malarial case misdiagnosis, the infection can re-emerge. An impressive lesson demonstrating a re-emerging infection was observed in Greece, where a vivax malaria outbreak occurred in 2009 after malaria was declared eliminated in 1973 [23], in addtion, more countries including Italy, Cyprus and Costa Rica have reported the re-emerging infection recently (https://www.cdc.gov/malaria/).

In China, a public health system network has been established and covers public health from the national to the most basic levels in rural villages. For example, each village has a village clinic with at least one doctor, where patients suspected of having malaria are advised to transfer to hospitals for parasite assessment and treatment as soon as possible, and there are specific sections/departments that are responsible for public health care, including malaria control and elimination, at health care institutions at the township and upper governmental levels. However, a lack of sufficient technology and ability has always affected the staff in malaria-associated departments, especially at lower levels. Furthermore, because the number of malaria cases is decreasing, less attention or financial support is directed to malaria control and elimination, making disease control worse at a basic level. In this study, there was a relatively high percentage of parasite species corrected by a provincial microscopy center from the cases that were reported by the sub-provincial organizations in Jiangsu in 2013 and 2014 (Table 1). In addition, many patients (23.14%) went to township and village hospitals but did not receive a confirmed diagnosis of malaria (Fig. 4), which indicates that capacity for diagnosis, including microscopy examination skills, should be improved.

The quality of microscopic malaria examination is dependent on the competence and performance of laboratory technicians, including blood smear preparing, staining, and interpreting. In this study, staff with higher education levels were more likely to be found at upper county levels than in townships. This study shows that staff at a county level, including county CDCs and county hospitals, exhibited much better skills than staff from township hospitals or PHCs in terms of parasite interpretation. In fact, a small percentage of the staff at township hospitals and PHCs could not differentiate falciparum from other species, which could place the current malaria elimination surveillance system at risk. In addition, the ability to prepare slides was also closely related to educational background. For example, microscopists with a bachelor degree had better blood smear preparation skills than those who graduated from a 2-year specialized secondary school. Consequently, it is imperative to encourage more young and promising graduates with a higher education level to join the malaria control and elimination network.

In elimination settings, a village clinic doctor or township PHC workers are responsible for malaria case management and subsequent investigations. In this study, a substantial number (more than 20%) of the malaria patients went first to the township or lower level to seek medical treatment because of febrile or other malaria-related symptoms. However, none of these patients received a confirmed diagnosis at the township and village level. This inability to diagnose malaria might represent the situation in the entire province, because similar results were found from reporting data in other cities in Jiangsu Province: a zero or a very low percentage of patients received malaria confirmation at the township or village level. The main reason for this lack of confirmation might be due to inadequate ability in the lower-level staff to microscopically distinguish plasmodium parasites from artifacts. In addition, because the staff in township hospitals in particular are responsible for many disease diagnoses and treatments, it is difficult to perform well under time constraints and a heavy workload, and poor quality blood smear preparation might result. Accordingly, periodic refresher training, frequent supervision, and the establishment of a testing program should be provided by a provincial reference laboratory to lower level personnel, especially in townships in the province, to maintain microscopic skills and ability. Additionally, an alternative approach, such as RDTs, should also be considered for use, particularly at the township and village levels, to avoid potential misdiagnoses and missed diagnoses of malaria, which may cause death in falciparum malaria cases due to the lack of timely and appropriate diagnosis and treatment.

## Conclusions

The insufficient capacity for malaria diagnosis at a lower level is one of the challenges to achieving and maintaining the goal of malaria elimination in China. Therefore, regular training and supervision of microscopic examination skills should be provided to staff especially at the township level. In addition, more graduates with a higher degree should be encouraged to join the public health network to improve the current township capacity for malaria elimination. Moreover, an alternative approach, such as using RDTs, should supplement microscopy examination especially at a lower level.
